# Gender Identity Development among Teenagers Living in the Subarctic Region of Russia

**DOI:** 10.3390/bs8100090

**Published:** 2018-09-29

**Authors:** Natalia Flotskaya, Svetlana Bulanova, Maria Ponomareva, Nikolay Flotskiy, Tatiana Konopleva

**Affiliations:** Higher School of Psychology, Pedagogy and Sport Teachers’ Training, Northern (Arctic) Federal University named after M.V. Lomonosov, 163002 Arkhangelsk, Russia; n.flotskaya@narfu.ru (N.F.); s.bulanova@narfu.ru (S.B.); m.ponomareva@narfu.ru (M.P.); flotskij.ns@edu.narfu.ru (N.F.)

**Keywords:** gender identity, gender identity types, teenagers

## Abstract

Gender identity is an important element of an individual’s identity and is one of the regulators of human behavior while acquiring social roles. The aim of this empirical research is to study gender identity development among teenagers living in the subarctic territories of Russia. The results show the correlation between types of gender identity among male and female teenagers, the dynamic in the correlation between gender identity types during adolescence and the characteristics of each type of gender identity in adolescence from the point of view of psychological properties.

## 1. Introduction

Modern society is now at the stage of development characterized by extraordinarily pressing problems, which have to do with the self-identification of men and women. This is directly related to the development of the gender identity of an individual. The need for identity is a fundamental need of any individual that is essential for their mental health [1]. Gender identity as a key element of subjective reality has a direct relationship with society and is formed by social processes. Gender identity is a part of the dynamic structure of one’s personality and is one of the regulators of human behavior during acquisition of various social roles. Reference to a certain gender determines one’s interests and formation of a special system of ideas about oneself as a person. Gender identity determines to a great extent the system of values, motives and patterns of human behavior [2,3,4,5,6].

The momentum of social processes and constant emergence of new challenges that require a response and a high level of individual adaptive potential are characteristic of modern society. While interacting with the changing living conditions, an individual gets into a kind of an adaptive conflict: The need for self-preservation is not provided by outdated adaptation programs. It is known that gender identity is directly related to the level of an individual’s adaptive potential [7,8,9,10,11,12,13]. People with different types of gender identity are characterized by different adaptive capabilities. For a long time, there has been a theory in psychology that the masculine type of gender identity is best for men and the feminine type of gender identity is best for women [14,15,16]. However, nowadays this theory is no longer applicable. Contemporary society is so diverse and the social roles of men and women are so multifaceted that it would be ill-considered to define them only with the parameters of masculinity for men and femininity for women. The current situation is characterized by multipolarity and a blend and convergence of masculine and feminine characteristics of an individual. Each type of gender identity has its own unique set of psychological properties and characteristics. The question therefore arises: What is the difference between the personal qualities of people and various types of gender identity? We assume that each type of gender identity has its own set of psychological qualities. What do these sets consist of? In modern psychological research we did not find any data containing a complete list of psychological properties characteristic of any particular type of gender identity. We find only separate studies that give us a fragmentary image [17,18,19,20,21,22]. While knowledge of psychological qualities characteristic of one or another type of gender identity is important for proper organization of an individual’s socio-educational environment, we understand that the environment should be comfortable, humane and personality-oriented. Interactions between the subjects of the socio-educational environment should also be taken into account. All the aforementioned ensures the socialization of an individual and contributes to personal development in general. Therefore, the complete picture of psychological properties characteristic of each type of gender identity is important indeed.

The most significant phase of a gender identity acquisition is adolescence [13,23,24,25,26]. This is a period of social, emotional and cognitive development between childhood and maturity. This period is characterized by asynchrony of physiological and mental maturation, the mismatch between real experience and an increased need for self-assertion. It is also characterized by a growing sense of independence, personal autonomy and identity. The central and specific trait of the adolescent period is the sense of adulthood, which is based on physiological changes in one’s own body, as well as on the subjective perception of social changes. A sense of maturity expresses the teenager’s new life attitude towards oneself, other people, and the world and determines one’s social activity and specific features of one’s inner life [27]. In adolescence with the formation of a new level of self-awareness, people start to form a relatively stable image of oneself as a representative of a particular gender, an individual choice of sexual behavior and an “image” of one’s gender role [28]. All those make adolescence a sensitive period for one’s gender identity formation. Thus, the questions arise: Which types of gender identity are characteristic of adolescence and do any changes occur in the correlation of gender identity types or do they remain static throughout adolescence?

Having analyzed the aforementioned approaches we have come to form the following hypotheses:

1. We assume that there is a certain correlation between the types of gender identity that are different between males and females in adolescence.
2. We assume that during adolescence the correlation between gender identity types is not static, it is in the process of changing.
3. We assume that each type of gender identity in adolescence has a specific set of psychological properties.

An empirical test of these hypotheses showed that among teenagers there exists a certain correlation of gender identity types. This correlation is not the same for males and females, moreover, the correlation of gender identity types varies during adolescence. Each gender identity type is characterized by a certain set of psychological qualities.

## 2. Materials and Methods

### 2.1. Participants

In order to reveal special features of gender identity development and to analyze personal qualities characteristic of each gender identity type in adolescence, we have conducted a longitudinal research project consisting of three stages. During the first stage we chose 241 respondents aged 11–12 years old, among which there were 114 boys and 127 girls. All the respondents come from the subarctic area of European Russia (Arkhangelsk region). The teenagers who have taken part in the research were students in the 4th and 5th grades of secondary school.

The second stage of research was carried out three years after the first stage took place. 238 teenagers of those from the first stage took part in the second stage of the research, now at the age of 14–15. There were 112 teen boys and 126 teen girls. The teenagers involved were students in the 7th and 8th grades of secondary school at that time. 

The third stage of research was carried out three years after the second stage took place. For the third stage the same teenagers were involved. The total number of respondents was 224 people, 105 of which were teen boys and 119 were teen girls. At this stage of the research the teenagers were at the age of 17–18 and were students in the 10th and 11th grades of secondary school. The decrease in the number of the respondents was caused by their moving away, absence due to illness, etc. Thus, the sample of the re-selection was not statistically different from the initial one. 

### 2.2. Procedure

The territories of European Russia (Arkhangelsk region) received the status of the subarctic territories, which made it necessary to obtain additional information about the psychology of the younger generation from these territories. The research was carried out on the initiative of a group of scientists from the Northern (Arctic) Federal University. 

During the organizational stage of the research we prepared a set of documents consisting of topical justification, description of its aims, objectives and stages and characteristics of research methods. This set of documents was submitted to the Ministry of Education and Science of the Arkhangelsk region. As a result, we received authorization to conduct the research.

A number of secondary schools in the Arkhangelsk region were chosen to serve as a research base. We met the principals of each participating secondary school and explained the aims, objectives and the procedure of the research to them. The principals helped us to make the selection of particular forms for the research. Together with the form masters we scheduled meetings with the respondents. The research was carried out in groups of 10–12 people, with 2 meetings held with each group. Each meeting lasted for 45 min (one academic hour).

Before the research started we had held meetings with the parents of the respondents. We explained the aims, objectives and procedure of the research to the parents. The parents and legal representatives of the students gave us a written consent to the students’ participation in the research.

In accordance with the schedule groups of students gathered in the classroom. They started with writing their age and answering a question “Who do you identify yourself with: A boy or a girl?” on the distributed sheets of paper. Then they received the test forms. The researcher gave instructions to the students and they filled in the answer sheets. If the respondents had questions the researcher approached them and individually gave the necessary explanations.

The results of the research were then reported to the teenagers’ parents, school psychologists and teachers. 

### 2.3. Methods

Two questionnaires were used in the research: Bem Sex-Role Inventory (BSRI) [29] and Raymond B. Cattell’s Sixteen Personality Factor Questionnaire [30].

With the help of BSRI we have defined the gender expression of masculinity and femininity among the males and females on various stages of adolescence. With the gender expression of masculinity and femininity as a basis we have defined types of gender identity. The procedure for carrying out this method was as follows. The respondents were given a list of 60 different personality traits consisting of masculine, feminine and gender neutral traits. The participants were asked to rate themselves on each trait using a 7-point Likert scale. Then we counted each respondent’s total amount of received points using the masculinity and femininity scales. Individual scores of each respondent was compared with the median calculated for the age group which the respondent belonged to. A score equal to or above the median were considered to be high. The score below the median was considered to be low. We looked at the ratio of masculine and feminine categories of each respondent and defined their type of gender identity. These types included androgynous gender type (high masculine score—high feminine score), masculine gender type (high masculine score—low feminine score), feminine gender type (high feminine score—low masculine score) and undifferentiated gender type (low masculine score—low feminine score).

With the help of Raymond B. Cattell’s Sixteen Personality Factor Questionnaire we studied personal characteristics of the representatives of each type of gender identity. In our research we used the questionnaire C—a shortened version consisting of 105 questions. The questionnaire (C) contains statements which have to do with behavior, emotional states, orientations and attitudes to life’s difficulties and is to test personal traits. We have diagnosed the following factors: A—“warmth”, B—“reasoning”, C—“emotional stability”, E—“dominance”, F—“liveliness”, G—“rule-consciousness”, H—“social boldness”, I—“sensitivity”, L—“vigilance”, M—“abstractedness”, N—“privateness”, O—“apprehension”, Q1—“openness to change”, Q2—“self-reliance”, Q3—“perfectionism”, Q4—“tension”. Respondents were given detailed instructions on how to work with the questionnaire. They chose the answer option which most matched their point of view. Then we processed the received data with the help of a special “key”. The received “raw” scores were converted into stans. 

### 2.4. Statistical Analysis

The data was processed with the help of SPSS Statistics 22. In order to test the second hypothesis we used Pearson’s chi square test. With the help of this method we detected considerable differences in the breadth of this or that type of gender identity among the respondents of different ages. To test the third hypothesis we used the method of correlation analysis and r-Pearson’s correlation coefficient. We detected presence and direction of dependencies between masculinity and femininity and personal qualities of male and female teenagers.

## 3. Results

Our first hypothesis was that there is a certain correlation between the types of gender identity that is different between males and females in adolescence. The distribution of gender identity types among male and female teenagers is shown in Table 1. The analysis of gender identity types distribution among boys and girls was conducted by non-statistical comparison. 

Our second hypothesis was that during adolescence, the correlation between the types of gender identity is not static; it is in the process of changing. The distribution of gender identity types during different stages of adolescence is shown in Table 1.

Statistical analysis showed that the occurrence of androgynous gender type among the boys falls considerably from age 11–12 to 17–18 (*p* < 0.01). We found the tendency for undifferentiated (*p* < 0.05) and feminine (*p* < 0.05) gender types to increase their occurrence among the boys from 11–12 to 17–18 years old.

Statistical analysis showed that the occurrence of feminine gender type among girls increases considerably from age 11–12 to 17–18 (*p* < 0.01) while the occurrence of undifferentiated gender type falls (*p* < 0.01) under the same age period.

Our third hypothesis was that each type of gender identity in adolescence has a specific set of psychological properties.

Correlation dependencies of masculinity and femininity expressions with Raymond B. Cattell’s Sixteen Personality Factor Questionnaire among male and female teenagers are shown in Table 2 and Table 3.

As Table 2 shows, masculinity and femininity expressions among male teenagers with masculine type of gender identity correlate with such expressions as rule-consciousness, social boldness, sensitivity, apprehension, openness to change and tension. Masculinity and femininity expressions among male teenagers with feminine type of gender identity correlate with such expressions as emotional stability, liveliness, abstractedness, privateness, openness to change and self-reliance. Masculinity and femininity expressions among male teenagers with androgynous type of gender identity correlate with such expressions as warmth, rule-consciousness, openness to change and perfectionism. Masculinity and femininity expressions among male teenagers with undifferentiated type of gender identity correlate with such expressions as warmth, dominance, rule-consciousness, social boldness, openness to change and perfectionism.

As Table 3 shows, masculinity and femininity expressions among female teenagers with masculine type of gender identity correlate with such expressions as warmth, dominance, social boldness, vigilance, privateness and apprehension. Masculinity and femininity expressions among female teenagers with feminine type of gender identity correlate with such expressions as sensitivity, vigilance and privateness. Masculinity and femininity expressions among female teenagers with androgynous type of gender identity correlate with such expressions as dominance, social boldness and perfectionism. Masculinity and femininity expressions among female teenagers with undifferentiated type of gender identity correlate with such expressions as sensitivity, vigilance, openness to change and perfectionism.

## 4. Discussion

First of all it should be noted that 100% of our respondents identified their gender in accordance with their biological sex. The analysis of results has made it possible for us to answer the questions formulated in our hypotheses. We assumed that there is a certain correlation between the types of gender identity that is different between males and females in adolescence. At the age of 11–12 the most widespread type of gender identity among boys and girls is the androgynous gender type. This type of gender identity presupposes the occurrence of masculine and feminine qualities, which are expressed to a high extent [9]. Regarding boys the most widespread types of gender identity among the remaining ones are the masculine and undifferentiated gender types. In our opinion, the “masculine type” phenomenon doesn’t need any additional explanations. As for the “undifferentiated type” of gender identity, let us note that this type implies the occurrence of masculine and feminine qualities, but they are subtle [11]. Regarding girls the most widespread types of gender identity among the remaining ones are the feminine and undifferentiated gender types. The type that occurs least among boys is the feminine gender type. The type that occurs least among girls is the masculine gender type. To sum up, we can say that at the age of 11–12 the correlation between the types of gender identity is different among boys and girls. 

In the next group of the respondents (boys and girls at the age of 14–15) the androgynous type of gender identity prevails, but regarding boys it is the androgynous type of gender identity that is most widespread among the remaining ones, then the masculine type and the least prevailing type of gender identity is the feminine one. Regarding girls it is the feminine type of gender identity that is the most widespread among the remaining ones. The least dominant types of gender identity are the masculine and undifferentiated ones. Thus, at the age of 14–15 we again see differences in the correlation of the gender identity types among boys and girls.

At the age of 17–18 the prevailing types of gender identity among boys are the undifferentiated and androgynous ones and the prevailing types of gender identity among girls are the androgynous and feminine ones. At the same age period it is the masculine type of gender identity that dominates among the remaining ones (masculine and feminine types) among boys. When we talk about girls the remaining types of gender identity (masculine and undifferentiated types) are less dominant. Thus, at the age of 17–18 we find differences in the correlation of the gender identity types among boys and girls.

As such, our findings have supported our first hypothesis. We have found that there is a certain correlation between gender identity types among teenagers. This correlation varies among males and females. We have come across this idea in other studies as well [23,31]. We have found that the most prevailing types of gender identity among male teenagers are the androgynous, undifferentiated and masculine ones. The most prevailing types of gender identity among female teenagers are the androgynous and feminine ones. Thus, we can say that the least prevailing type of gender identity among both boys and girls is the one that is not close to their gender (feminine type for boys and masculine one for girls). It should be noted that the undifferentiated type of gender identity is more widespread among boys compared with girls.

Our next hypothesis was that the correlation between the gender identity types is not static during adolescence, it keeps changing. We have come across this point of view in a number of studies [13,23,24,26,32,33]. In fact, our research has shown that the correlation between gender identity types among boys does change during adolescence. At the age of 11–12 and 14–15 the most prevailing type of gender identity among boys is the androgynous one. At the age of 17–18 two types of gender identity become prevalent among boys. They are the undifferentiated and androgynous types. We want to emphasize the fact that during adolescence the occurrence of the androgynous type of gender identity among boys falls considerably. There is a tendency for some increase in the undifferentiated and feminine types of gender identity. We think that such a situation with the correlation of gender identity types among boys has to do with general processes of socializing typical of the subarctic territories of European Russia. At the age of 17–18 teen boys have sufficiently developed self-awareness and their mindset. They start to experience feelings of maturity. The majority of teen boys still go to school or college, remain financially dependent on their parents and live with them. However, it is very common for people from these territories to think of a man as self-sufficient, financially independent and successful at work. This is why boys have quite limited opportunities to try various sex-role models.

The occurrence of the androgynous and masculine types of gender identity among girls lies almost on the same level during adolescence. It should be noted that from age 11–12 to 17–18 the occurrence of the feminine type of gender identity grows considerably while the occurrence of the undifferentiated type falls. In our opinion, such a situation with the correlation of gender identity types among girls has to do with general processes of socializing typical of the subarctic territories of the European Russia. Historically, the gender-based division of labor in the subarctic territories of European Russia was affected by severe weather conditions, traditional lifestyle patterns and traditional crafts. In these territories it is common to think of a woman as a wife or a mother. Furthermore, financial independence and professional self-realization are not so important when one measures her successfulness. That is why by the age of 17–18 girls have had more opportunities to realize the feminine sex-role model. Thus, our second hypothesis has proved to be true. We have proved our assumption that the correlation between gender identity types keeps changing during adolescence.

Another hypothesis concerned our assumption that each type of gender identity during adolescence has a specific set of psychological properties. We haven’t found in other studies a full list of psychological properties characteristic of each type of gender identity. There are a few works which describe some psychological properties characteristic of one or another gender identity type [17,18,19,20,21,22].

The analysis of results of our research has proved that there is a correlation between femininity and masculinity and various personal qualities in each gender identity type of boys and girls. As a result we have managed to single out sets of psychological properties characteristic of each gender identity type.

Male teenagers with the masculine type of gender identity usually have qualities such as self-confidence, courage, rigidity, radicalism, low rule-consciousness and tension. Female teenagers with the masculine type of gender identity usually have such qualities as reserve, dominance, vigilance, straightness and self-confidence. 

Male teenagers with the feminine type of gender identity usually have qualities such as emotional instability, liveliness, abstractedness and diplomacy. Female teenagers with the feminine type of gender identity usually have such qualities as sensitivity, credulity and diplomacy. 

Male teenagers with the androgynous type of gender identity usually have qualities such as sociability, rule-consciousness, radicalism and perfectionism. Female teenagers with the androgynous type of gender identity usually have such qualities as dominance, social boldness and perfectionism. 

Male teenagers with the undifferentiated type of gender identity usually have qualities such as reserve, dependence, impulsiveness, timidity, conservatism and low self-control. Female teenagers with the undifferentiated type of gender identity usually have qualities such as rigidity, vigilance, radicalism and low self-control.

Thus, our third hypothesis has proved to be true. We have defined the sets of psychological properties characteristic of each gender identity type in adolescence.

## 5. Conclusions

The results of our research have theoretical and practical value. Adolescence is a sensitive period for gender identity development [13,23,24,25]. At the same time, it is difficult for teachers to influence the process of personal development during this period. The results of our study will help teachers and psychologists who work with adolescents to find an individual approach to the adolescents from the subarctic territories of European Russia. Understanding the psychological properties characteristic of each type of gender identity will help to build relationships with adolescents and to predict a trajectory of their individual development. The information about changes in gender identity throughout the adolescent period, presented in the results of our study, will help to plan and make necessary adjustments in the social and educational environment of adolescents in time.

All things considered, one’s knowledge of peculiarities of gender identity development in adolescence as well as understanding of the psychological properties characteristic of each type of gender identity are necessary for effective interaction with adolescents in the process of education and upbringing.

## Figures and Tables

**Table 1:** Distribution of gender identity types among the boys and girls at 11–12, 14–15 and 17–18 years old.

Gender Role Group	Male, n (%)			Female, n (%)		
	11–12 y.o.	14–15 y.o.	17–18 y.o.	11–12 y.o.	14–15 y.o.	17–18 y.o.
Masculine	23 (20,18)	17 (15,18)	25 (23,81)	18 (14,17)	19 (15,08)	17 (14,29)
Feminine	6 (5,26)	11 (9,82)	16 (15,24)	27 (21,26)	37 (29,37)	41 (34,45)
Androgynous	57 (50)	55 (49,11)	30 (28,57)	50 (39,37)	50 (39,68)	46 (38,66)
Undifferentiated	28 (24,56)	29 (25,89)	34 (32,38)	32 (25,20)	20 (15,87)	15 (12,60)
Total	114 (100)	112 (100)	105 (100)	127 (100)	126 (100)	119 (100)

**Table 2:** Correlation dependencies of masculinity and femininity expressions with Raymond B. Cattell’s Sixteen Personality Factor Questionnaire among male teenagers.

Gender Identity Type	Masculinity/Femininity	A	B	C	E	F	G	H	I	L	M	N	O	Q_1_	Q_2_	Q_3_	Q_4_
1	2	3	4	5	6	7	8	9	10	11	12	13	14	15	16	17	18
Masculine	M							* 0.29									* 0.30
	F						** 0.43		* −0.30				* −0.27	* −0.37			
Feminine	M													* 0.30	* −0.33		
	F			* −0.25		* 0.27					** 0.35	* 0.29					
Androgynous	M													* 0.36		** 0.40	
	F	* 0.24					* 0.27										
Unidentified	M				** 0.37		* 0.15	** 0.31								* 0.31	
	F	* 0.25												* 0.30			

Note: A—“warmth”, B—“reasoning”, C—“emotional stability”, E—“dominance”, F—“liveliness”, G—“rule-consciousness”, H—“social boldness”, I—“sensitivity”, L—“vigilance”, M—”abstractedness”, N—“privateness”, O—“apprehension”, Q1—“openness to change”, Q2—“self-reliance”, Q3—“perfectionism”, Q4—“tension”; * *p* < 0.05; ** *p* < 0.01.

**Table 3:** Correlation dependencies of masculinity and femininity expressions with Raymond B. Cattell’s Sixteen Personality Factor Questionnaire among female teenagers.

Gender Identity Type	Masculinity/Femininity	A	B	C	E	F	G	H	I	L	M	N	O	Q_1_	Q_2_	Q_3_	Q_4_
1	2	3	4	5	6	7	8	9	10	11	12	13	14	15	16	17	18
Masculine	M	* −0.32			* 0.28			* 0.26				* −0.26					
	F									** −0.34			* 0.27				
Feminine	M								** −0.36	** 0.34							
	F											* 0.24					
Androgynous	M				* 0.25			* 0.22									
	F															* 0.27	
Unidentified	M								* 0.24					* −0.27		* 0.34	
	F									* −0.28							

Note: A—“warmth”, B—“reasoning”, C—“emotional stability”, E—“dominance”, F—“liveliness”, G—“rule-consciousness”, H—“social boldness”, I—“sensitivity”, L—“vigilance”, M—“abstractedness”, N—“privateness”, O—“apprehension”, Q1—“openness to change”, Q2—“self-reliance”, Q3—“perfectionism”, Q4—“tension”; * *p* < 0.05; ** *p* < 0.01.
